# An analysis of the utilisation of medical identification jewellery among children and young adults with type 1 diabetes mellitus in Australia

**DOI:** 10.1007/s12020-022-03224-3

**Published:** 2022-11-05

**Authors:** Madeleine Heath, David J. Torpy, Rosemary Louise Rushworth

**Affiliations:** 1 School of Medicine Sydney, The University of Notre Dame, Australia, Sydney, NSW Australia; 2 Endocrine and Metabolic Unit, Royal Adelaide Hospital, Adelaide, SA Australia; 3 University of Adelaide, Adelaide, SA Australia

**Keywords:** Diabetes, Diabetes mellitus, Hypoglycaemia, Hyperglycaemia, Diabetic ketoacidosis, Medical identification jewellery

## Abstract

**Aims:**

People with type 1 diabetes mellitus (T1DM) are at risk of life-threatening illness. Medical jewellery is recommended for emergencies, but its uptake is unknown. This study assessed the use of medical jewellery among people with T1DM aged 0–24 years in Australia.

**Methods:**

A cross sectional analysis of subscription data to the largest medical identification jewellery service in Australia was analysed by age, sex and geographic location using Australian population data from 2018.

**Results:**

There were 1599 people with T1DM aged 0–24 in the database, but only 1061 had an active subscription, corresponding to an active subscription rate of 13.28/100,000 population or ~5% of the estimated patient population. Half of the active subscribers were male (543/1061, 51/3%). The average age of active subscribers was 17; very few (*n* = 12, 1.1%) were aged less than 5; and the highest number (*n* = 141, 39%) was in the 20–24 age group. Active subscription rates varied significantly by geographic location. 88.4% of active subscribers had a diagnosis of T1DM or equivalent inscribed on their emblem, while engraved instructions for management in an emergency were only included in 1.8% of records (*n* = 19).

**Conclusions:**

Medical jewellery subscription rates were lower than expected; increased with age; and varied significantly by state/territory. The use of medical identification jewellery may be limited by the lack of suitable engraved instructions for use in an emergency. Factors leading to low use should be addressed.

**Figure:**
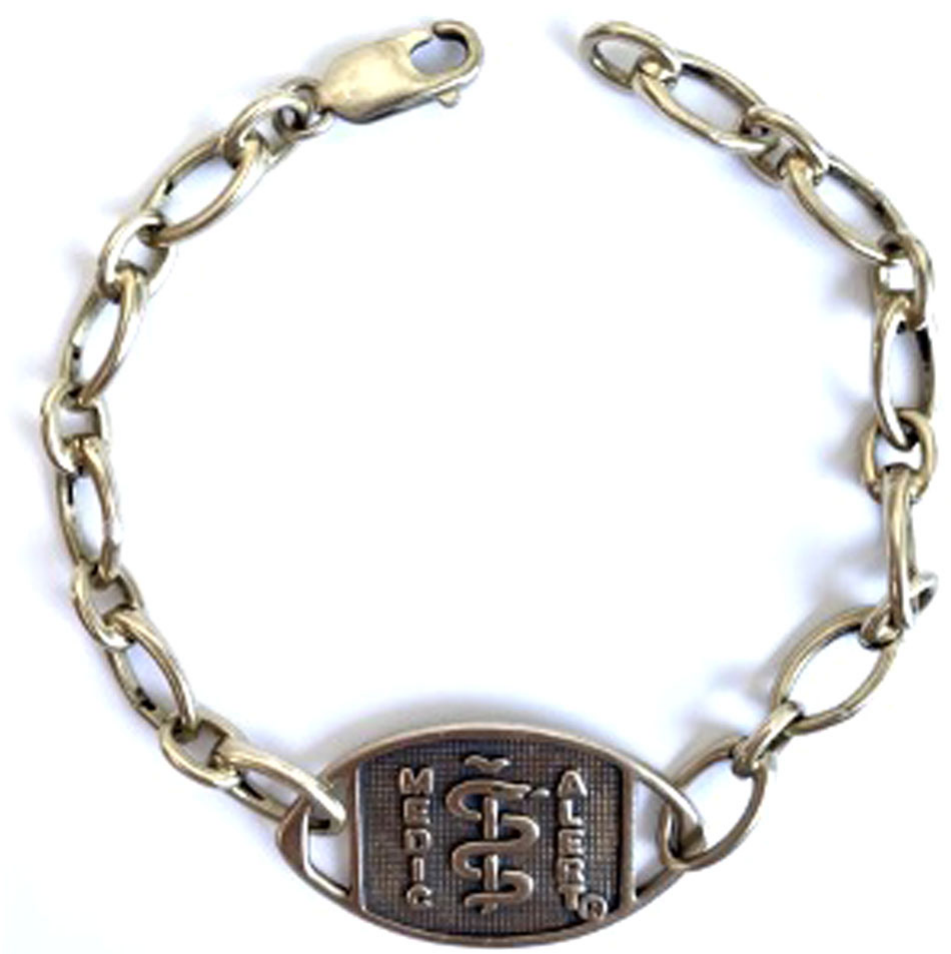
**Photo 1** Medical Jewellery with emblem

## Introduction

Diabetes mellitus (DM) is a chronic metabolic disorder that is characterised by abnormal carbohydrate metabolism and chronic hyperglycaemia. Elevated blood glucose levels are either the result of defective insulin production, defective insulin action or both [1]. Type 1 diabetes mellitus (T1DM) accounts for ~10% of all cases of diabetes in the population and 60% of all new cases are diagnosed in people aged under 25 years [2]. This corresponds to an estimated 20,700 Australians aged 0–24 years living with T1DM [2].

People with T1DM are at increased risk of morbidity and mortality from both acute and long-term exposure to abnormal blood glucose levels [3–5]. In those aged under 25, acute episodes of hypoglycaemia, hyperglycaemia and ketoacidosis are an important cause of morbidity and occasional mortality [3]. Hypoglycaemia, which is typically the result of both therapeutic hyperinsulinemia and compromised physiological and behavioural defences against falling plasma glucose concentrations, is the most feared complication among diabetic people [6] and accounts for 10% of deaths of people with T1DM [7, 8]. It is characterised by neurogenic and neuroglycopenic symptoms including tremor, palpitations, diaphoresis, weakness, impaired cognition, and, at lower plasma glucose concentrations, seizures and coma [9–11]. Hyperglycaemic episodes, especially those associated with ketoacidosis, can result in focal neurological signs (hemianopia and hemiparesis), seizures and alterations in mental status including profound lethargy and coma [12].

Patients and their families are taught to monitor blood glucose levels, recognise the signs and symptoms of hypoglycaemia, and treat promptly with oral glucose or, in severe cases, intramuscular glucagon injection [13]. Likewise, during times of illness and/or in the setting of significant or prolonged hyperglycaemia, patients and families are taught to check for urinary or blood ketones and treat with insulin as required [13, 14]. While many instances of altered blood glucose levels are successfully self-managed, severe hypoglycaemia requiring assistance of prehospital emergency medical services is increasingly common [15]. In these circumstances, the patient or their carer may be unable to self-administer treatment or communicate a diagnosis of T1DM to medical attendants in an emergency, which may delay appropriate critical management. Hence, it is advised that patients wear medical identification jewellery, typically an inscribed pendant with a recognisable medical emblem, to avoid delay in identification of the patient’s diabetes and suitable intervention by first responders should an emergency arise [16–18].

The major medical jewellery provider in Australia operates a subscription service, whereby patients pay an initial enrolment fee followed by an annual subscription and receive a pendant engraved with a unique MedicAlert number on which they can inscribe relevant medical information (Photo 1). Unique to this provider, the subscription also offers access to a 24 h telephone response service, which healthcare workers may ring, quote the identifying number on the medical jewellery, and obtain relevant details pertaining to the person’s medical history and treatments. Telephone access to medical records is only available to those with ‘active’ subscriptions. Those whose subscription has lapsed are considered ‘inactive’ and, although the person may continue to wear the jewellery, telephone access is unavailable.

The rate of usage of medical jewellery amongst children and young adults with T1DM is unknown. As such, this study aims to determine the uptake of medical identification jewellery by people with T1DM, and, given utilisation rates have been shown to be amenable to targeted interventions, to identify potential areas for improvement [19].

## Methods

The demographic and disease profiles of all subscribers to Australia’s predominant medical identification jewellery service (MedicAlert) are stored in a central database at the organisation’s headquarters in Adelaide, South Australia. Data are stored according to subscriber’s unique MedicAlert number, and subscriber records are comprised of all medical diagnoses; age; sex; state/territory of residence; medication and other treatments e.g. use of an insulin pump; whether a patient’s death was reported; and whether the subscription is active (current and up to date), lapsed (active subscription has ceased), or cancelled. Records also include details of any text inscribed on the jewellery, such as “Type 1 Diabetes: On Insulin”.

For this study, all eligible records for people aged 24 years and under were identified in the provider’s database by searching for the following diagnoses/terms: *diabetes*, *type 1, insulin, T1DM, glucose*. Once eligible patients were identified, the organisation’s staff de-identified the data and downloaded it into a separate dataset for analysis. The data extraction occurred in June 2020.

Permission to conduct this study was given by the Board of MedicAlert and by the Human Research Ethics Committee of the University of Notre Dame, Australia (HREC Reference Number: 2020124S).

### Data management

The study sample included records with a diagnosis of T1DM only. The record of one deceased person was excluded. People with T1DM were identified by either stipulation of a diagnosis or by information provided in either the medication or engraving text (e.g. insulin dependent diabetes mellitus). If not specified, diabetic patients treated with insulin were assumed to have T1DM in this age group.

### Data analysis

Rates of diabetics subscribed to the service were calculated using T1DM prevalence estimates published by the Australian Institute of Health and Welfare for residents aged 0–24 years in 2018 [2]. State and territory subscription rates were calculated using T1DM prevalence estimates for people aged 0–29 years from the National Diabetes Services Scheme (NDSS) in Australia [20]. Diabetes prevalence data for those aged 0–9 in the Northern Territory and Australian Capital Territory were unavailable. Apart from the overall subscription rates, which were presented as per 100,000 people in the general population, all other rates were presented as per 1000 estimated diabetics in each category. Subscription data from the Northern Territory and Tasmania were combined due to small numbers. Records from one person whose age was unknown and twelve whose sex was withheld were excluded from age and sex specific analyses of utilisation respectively.

Statistical analysis was performed using Excel (Microsoft Excel for Mac 2021) and SPSS Version 28.01. Patient age was categorised into 5-year age groups. Subscription rates were calculated using the relevant population for each region, age group and sex. Z scores were calculated to determine differences in subscription rates between the highest and lowest groups. A *p* value of <0.05 was considered significant.

## Results

There were 1599 children and young adults with a diagnosis of T1DM enroled in the database. This corresponds to an overall subscription rate of 20.01/100,000 people in this age group, which is ~7% of the estimated 259.00/100,000 Australian’s with T1DM aged 0–24.

### Active subscriptions

1061 (66.4%) of the children and young adults enroled in the database had an up to date (active) subscription, corresponding to an active subscription rate of 13.28/100,000 people in this age group, or ~5% of the estimated 259.00/100,000 people with T1DM aged 0–24.

**Fig. 1:**
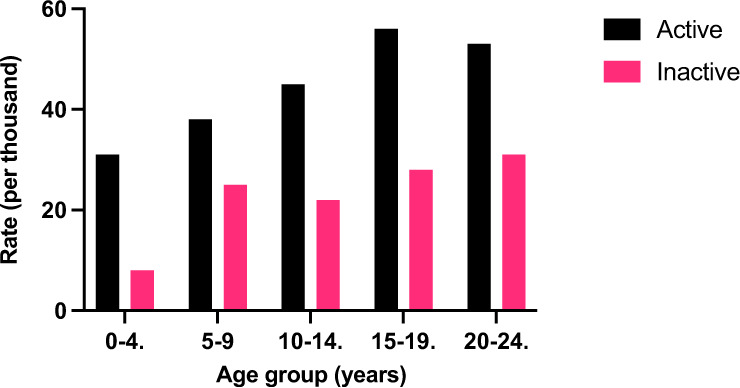
Rates of medical jewellery use by age group and subscription status, Australia 2020

Half of the active subscribers were male (543/1061, 51.3%) (Table 1). The mean age of active subscribers was 17.09 (SD = 5.0) years; very few (*n* = 12, 1.1%) were aged in the 0–4 age group; and the highest number (*n* = 414, 39.0%) was in the 20–24 age group (Fig. 1).

**Table 1:** Demographic and disease characteristics of active medical jewellery subscribers in Australia, 2020

State^a^	*N*	%	Rate^b^
NSW	197	12.5	23.04
Victoria	317	20.0	42.38
Western Australia	469	29.6	153.77
Queensland	134	8.5	21.00
South Australia	389	24.6	178.44
Australian Capital Territory	61	3.9	110.91
Other	15	0.9	16.85
Sex^c^			
Male	543	51.3	49.64
Female	515	48.7	51.63
Age group (years)^d^			
0–4	12	1.1	30.85
5–9	77	7.3	37.78
10–14	202	19.1	45.18
15–19	353	33.4	56.41
20–24	414	39.1	53.36
Insulin^e^			
Insulin Aspart (Novorapid)	507	47.8	
Insulin Detemir (Levemir)	89	8.4	
Insulin Lispro (Humalog)	88	8.3	
Insulin Glargine (Optisulin)	260	24.5	
Insulin Isophane Human (Protaphane)	36	3.4	
Comorbidities			
Asthma	69	6.5	
Coeliac Disease	60	5.7	
Thyroid Disease	24	2.3	
Autism	19	1.8	
ADHD	11	1.0	
Anxiety/Depression	18	1.7	
Engraving Text^f^			
‘Diabetes’	938	88.41	
‘On insulin’	893	84.17	
‘Call *emergency contact*’	9	0.8	
‘Call 000/ambulance’	6	0.6	
‘Give Sugar’	4	0.4	

^a^Includes patients aged 0–29

^b^Rate as per 1000 diabetic patients in each group

^c^Gender (*n* = 1058), excludes missing

^d^Age Group (*n* = 1061), excludes missing

^e^Some patients gave more than one

^f^Some patients gave more than one

**Fig. 2:**
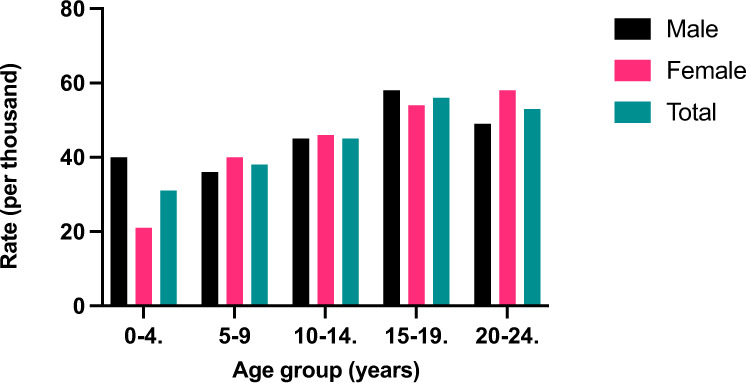
Rates of medical jewellery use by age group and sex, Australia 2020

There were substantial differences in the active subscription rates between the age groups and between the sexes within the age categories. In men, the lowest rate in was in the 5–9-year age group (36.1/1000) while the highest was in those aged 15–19 years (58.1/1000) (*P* < 0.00001) (Fig. 2). The lowest rate in females was in the 0–4-year age group (21.3/1000) whilst the highest was in the 20–24-year age group (58.0/1000) (*P* < 0.05) (Fig. 2).

**Fig. 3:**
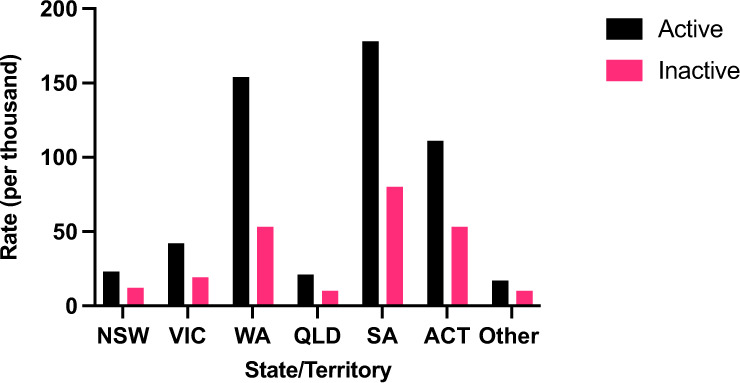
Rates of medical jewellery use by region and subscription status, Australia 2020. ACT Australian Capital Territory, NSW New South Wales, QLD Queensland, SA South Australia, VIC Victoria, WA Western Australia, Other Northern Territory and Tasmania

Active subscription rates varied between geographic areas. South Australia had the highest subscription rate (178.4/1000, 25.8%) which was sixteen times that of Tasmania where the subscription rates were the lowest (11.0/1000, 1.6%) (*P* < 0.00001) (Fig. 3).

Among all active subscribers, the most frequently recorded co-morbidity was asthma (*n* = 69, 6.5%), followed by coeliac disease (*n* = 60, 5.7%) and thyroid disease (*n* = 24, 2.3%). Insulin therapy was recorded in the medical history of 78% (*n* = 828) of active subscribers, with 21.9% of subscribers citing use of an insulin pump (*n* = 232). The most recorded insulin was insulin aspart (Novorapid) (*n* = 507, 46.8%), followed by insulin glargine (Optisulin) (*n* = 260, 24.5%).

88.4% of active subscribers (*n* = 938) had a diagnosis of Type 1 Diabetes Mellitus or equivalent inscribed on their emblem, and 84.2% (*n* = 893) stated use of insulin. Engraved instructions in the setting of an emergency were only included in 1.8% of records (*n* = 19), with most of those providing phone details of an emergency contact (*n* = 9), followed by a request to contact emergency services (e.g. 000 or ‘ambulance’) (*n* = 6) and instructions to give sugar (*n* = 4). No inscriptions mentioned the giving of glucagon.

### Inactive subscriptions

Of the subscriptions, 33.7% were classified as inactive (*n* = 539), corresponding to a rate of 6.73/100,000 people in this age group in the population, or ~2% of the estimated 259.00/100,000 people with T1DM in this age group. The average age of patients whose subscription was inactive was 17.1 (SD = 4.9) years. In all age categories, there were fewer inactive subscribers than active (Fig. 1). Females comprised 50.8% (*n* = 273) of the inactive category. The highest proportion of inactive subscribers were aged 20–24 (*n* = 203, 37.8%) (Fig. 1).

The highest inactive subscription rate was in South Australia (79.8/1000) and lowest was in Tasmania (5.5/1000) (*P* < 0.05) (Fig. 3). However, the ratio between active and inactive subscriptions was highest in Western Australia (2.91) followed by Victoria (2.26) and South Australia (2.24), while the lowest ratio was found in the Northern Territory (1.4).

## Discussion

This is the first study to analyse subscription rates to a large medical identification jewellery service in children and young adults with T1DM. Based on national prevalence estimates, utilisation was very low, with only 7% of the estimated people with T1DM aged 0–24 in Australia having a subscription (comprising 5% active and 2% inactive) to the largest and physician-preferred provider. Subscription rates varied by age and geographic region, suggesting that medical advice and patient factors play a key role in medical jewellery utilisation. The utility of the jewellery may be limited by extremely low (1.8%) rates of engraved instructions for management in the setting of an emergency.

Overall, there was little gender variation in active subscription rates, except for in those aged under 4 years and in young adults. In the very young, males held active subscriptions at double the rate of females. While the rate of active subscriptions fell in males during young adulthood, subscription rates in females increased. This may reflect a greater personal or social acceptance of the use of medical jewellery in females or suggest that females have closer contact with healthcare providers than males in this age group.

There was considerable variation in medical jewellery subscription rates by patient age. Subscriptions were infrequent amongst pre-school aged children, possibly reflecting the close parental oversight of diabetic children in this age group, and a subsequent belief that there would be little to gain from the use of a medical identification subscription and jewellery. Subscription rates increased throughout childhood and peaked among teenagers, likely reflecting parental efforts to ensure their child’s safety during what is often a time of increased independence and risk-taking behaviours.

By contrast, subscription rates fell slightly amongst males in the 20–24 year age group. This fall in adherence could be the result of several factors, including an inability or hesitancy to pay the subscription fee (AUD $52) in the context of increased financial responsibility; a lack of appreciation for the utility of the membership; social unacceptability of jewellery use; a decision to transfer to a different provider; or less frequent contact with a health professional or clinical team compared to the more intense input provided during childhood and teenage years [21, 22].

The impact of social factors in determining usage of medical identification jewellery is emphasised by the similar uptake patterns found in children and adolescents with adrenal insufficiency, where usage was low in those aged under 4 years, increased with age, peaked in teenagers, and dropped off significantly in early adulthood [23].

Young adulthood has long been recognised as a particularly vulnerable period for people with T1DM [21]. It is underscored by the transition from paediatric to adult healthcare, which is a time where people with T1DM are at high risk for loss to follow-up, poor glycaemic control and increased hospitalisations [23, 24]. Moreover, it is typically during these years that alcohol and other recreational substances become increasingly prevalent in social settings, which has been associated with impaired diabetes self-management and hypoglycaemia [25, 26]. For these reasons, medical identification jewellery could be of particular benefit in this age group and, given adherence rates have been shown to improve following in-clinic education programs in people with adrenal insufficiency, emerging adults with T1DM should be a focus for future interventions [19].

There were marked differences in subscription rates between states, with the highest rates of active membership reported in South Australia and Western Australia. The geographical variations in subscription rates may also reflect a local relationship between clinicians and the provider, as the head office was initially based in Western Australia and is now based in South Australia. Notably, a similar pattern of uptake in Western Australia and South Australia was found in both the adult and paediatric populations of people with adrenal insufficiency, emphasising the impact of local factors in uptake, irrespective of the disease [22, 23]. It may be that variations in practice among diabetes physicians or diabetes educators between states underlie the very different rates of uptake and this is a factor that could be investigated. Similarly, it would be of interest if the use of MedicAlert is stimulated by experience of a diabetic emergency and associated hospital admission.

As of 2013, 43% of children aged under 14 years utilised insulin pump therapy in Australia [24]. Current rates of usage are anticipated to have risen since this then, particularly in the context of the national Insulin Pump Program which has afforded increased access to government subsidised insulin pumps and consumables [24]. In this study, however, insulin pump therapy was only reported in the records of 21.9% of subscribers. This could be because not all insulin pump users reported their use – be it by accidental or intentional omission, or failure to update their subscription records following insulin pump commencement. It may also reflect a reduced fear of hypoglycaemia, and subsequent perceived need for medical identification jewellery, amongst patients using insulin pump therapy [25–29]. Similarly, there may also be a belief amongst insulin pump users that recognition of an insulin pump by emergency responders would sufficiently identify their diabetic status and hence negate the need for medical identification jewellery.

Hypoglycaemia and ketoacidosis are persistent potentially life-threatening complications of T1DM, and hence require timely recognition and treatment [30]. In the event that a person is unable to self-treat or communicate a diagnosis, medical identification jewellery serves to facilitate both prompt identification of diabetic status, and for the lay person, provision of basic management instructions [16, 31, 32]. In this study, most people had phrases like ‘diabetic on insulin’ engraved onto their emblem. Although this may be sufficient to alert trained emergency personnel to consider a glycaemic event as a potential cause of the person’s state, misinterpretation by the lay person could have fatal implications if, for example, insulin was to be administered in the setting of hypoglycaemia.

Although there are currently no guidelines as to the most appropriate inscription, instructions such as ‘diabetic give sugar or glucagon’ may be more appropriate in providing simple guidance to any non-medical emergency attendant and would carry minimal risk of harm if administered to a hyperglycaemic patient [33]. The emergence of nasal glucagon for hypoglycaemia resuscitation will make its administration by untrained individuals simpler and quicker so its inclusion on medical identification jewellery will be pertinent. Regulating the use of medical identification in Australia, including standardised inscription guidelines, would possibly improve both the safety and utility of medical identification jewellery [31].

Although the findings of this study provide important information on the usage patterns of medical identification jewellery in people with T1DM, there are some limitations. While the data for this study was extracted from the largest medical jewellery subscription service in Australia, it is only one of many providers offering to engrave jewellery (but without a 24 h telephone emergency service). In addition, all data obtained from the provider was patient-reported, meaning information may be inaccurate or out of date. Although active subscriptions represent current subscribers, they do not necessarily reflect correct usage (wearing jewellery at all times) which may vary between age and sex. Likewise, it is possible that those with lapsed subscriptions may continue to wear the jewellery in the absence of the telephone response service. The reasons for lapsed subscriptions are unknown, and could potentially be due to emigration overseas, converting to a different service provider, or, in a small number of people, unreported death. Moreover, in the absence of national data for the diabetic population in each state, the number of diabetics in each state was estimated using data from the NDSS. While not all diabetics are subscribed to the NDSS, given the incentives they provide to people with diabetes, including subsidised glucose monitoring products, insulin pen needles and pump consumables, it is assumed that it captures almost all Australian patients.

In conclusion, this is the first study to examine patterns of uptake and adherence to subscriptions for medical identification jewellery in people with T1DM aged 24 years and under. Communication of a diagnosis of T1DM and emergent glycaemic management is critical in preventing morbidity and mortality associated with hypo-and hyper-glycaemic episodes. In situations where patients are too unwell to communicate a diagnosis, or self-manage blood sugar levels, use of medical identification jewellery, with clear emergency instructions inscribed on the emblem, may be lifesaving. Our study, however, found that usage rates were very low, and that factors such as exposure to healthcare providers, local connections to medical identification jewellery services, and social and economic overlays may influence uptake, and should therefore be addressed as areas for improvement.

## Data Availability

Data that support the findings of this study are available on request from the corresponding author. The data are not publicly available due to privacy or ethical restriction.
